# A multi-center, single-blinded, randomized, parallel-group, superiority study to compare the efficacy of manipulation under anesthesia versus intra-articular steroid injection in the treatment of patients with frozen shoulder and a diagnosis of rotator cuff injury or tear by MRI: study protocol for a randomized controlled trial

**DOI:** 10.1186/s13063-023-07810-2

**Published:** 2023-11-27

**Authors:** Wuwei Song, Xiaoyu Guo, Xiang Wang, Jiacheng Yu, Wenyu Jiang, Chen Wei, Yuhao Zhao

**Affiliations:** Shi’s Center of Orthopedics and Traumatology (Institute of Traumatology, Shuguang Hospital), Shuguang Hospital Affiliated to Shanghai University of Traditional Chinese Medicine, Shanghai, China

**Keywords:** Adhesive capsulitis, Frozen shoulder, Rotator cuff injuries, Manipulation under anesthesia, Intra-articular steroid injection

## Abstract

**Background:**

Frozen shoulder (FS) is a common condition that can cause severe pain and limited range of motion in the shoulder joint. While intra-articular steroid injection has been shown to be an effective treatment for FS, manipulation under anesthesia (MUA) is an alternative treatment that has gained popularity in recent years. However, there is a lack of evidence regarding the effectiveness of MUA on FS patients with concomitant rotator cuff injury or tear. Though a few studies have shown that MUA is not associated with rotator cuff tears, and will not exacerbate the injury, more high-quality studies with bigger sample sizes are needed. Therefore, the aim of this multi-center, single-blinded, randomized, parallel-group, superiority study is to compare the efficacy of MUA versus intra-articular steroid injection in the treatment of FS patients with a diagnosis of rotator cuff injury or tear by MRI.

**Methods:**

A parallel, single-blinded, multi-center randomized controlled trial of 320 patients will be conducted at three hospitals of China. Eligible patients with frozen shoulder and rotator cuff injury or tear diagnosed by MRI will be randomly assigned to, in equal proportions, the manipulation under anesthesia group and the intra-articular steroid injection group via a central randomization system, undergoing a corresponding operation on day one and a sequent physical exercise for 14 days. The primary outcome is the comprehensive efficacy evaluation (total effective rate) and the change of Constant-Murley Score. Outcome assessors and data analysts will be blinded, and participants will be asked not to reveal their allocation to assessors.

**Discussion:**

This study aims to explore the superiority of manipulation under anesthesia in reducing pain and improving shoulder function in frozen shoulder patients accompanied with rotator cuff injury. To provide a scientific basis for the dissemination and application of manipulation under anesthesia, and a better knowledge for the role of MUA in the treatment of frozen shoulder accompanied with rotator cuff injury.

**Trial registration:**

Chictr.org.cn ChiCTR2200067122. Registered on 27 December 2022. ChiCTR is a primary registry of the World Health Organization International Clinical Trials Registry Platform (WHO ICTRP) network and includes all items from the WHO Trial Registration data set in Trial registration.

## Administrative information

**Table:** 

Title {1}	A Multi-center, Single-blinded, Randomized, Parallel-group, Superiority Study to Compare the Efficacy of Manipulation Under Anesthesia versus Intra-articular Steroid Injection in the treatment of patients with Frozen Shoulder and a diagnosis of rotator cuff injury or tear by MRI: study protocol for a randomized controlled trial
Trial registration {2a and 2b}.	Chictr.org.cn ChiCTR2200067122. Registered on 27 December 2022. https://www.chictr.org.cn/showproj.html?proj=187462
Protocol version {3}	Protocol version 1.0, which was revised on 11 November 2022.
Funding {4}	This work was supported by Shanghai Science and Technology Commission Project: Clinical study of TCM manipulation in the treatment of the Frozen Shoulder (22Y21920200)
Author details {5a}	Wuwei Song, Xiaoyu Guo, Xiang Wang, Jiacheng Yu, Wenyu Jiang, Chen Wei, Yuhao Zhao
Name and contact information for the trial sponsor {5b}	Shanghai Science and Technology Commission Contact address: 200 People's Avenue, Huangpu District, Shanghai, China (200003)
Role of sponsor {5c}	The funding body plays no role in the design of the study and collection, analysis, and interpretation of data and in writing the manuscript.

## Background and rationale{6a}

Frozen shoulder (FS), also known as “adhesive capsulitis” [1], “fibrotic capsulitis” [2], “primary idiopathic stiff shoulder” [3], and “contracture of the shoulder” [4], is a common shoulder concern manifesting in progressive loss of glenohumeral movements coupled with the painful, debilitating contraction of the shoulder [5, 6]. It is a retrogressive progress of the fibroproliferative tissue, and in spite of the dissections owing to the immunobiological advances in other diseases of this condition, the molecular pathophysiology underpinning the idiopathic FS is still poorly understood [7–10]. Idiopathic FS is characterized by a spontaneous onset of pain and stiffness of the shoulder, especially a loss of external rotation, without a prior traumatic event [11]. In May 2014, the International Society of Arthroscopy, Knee Surgery and Orthopedic Sports Medicine (ISAKOS) first proposed and published an expert consensus on frozen shoulder at the meeting held in Amsterdam which defined “frozen shoulder” as a limited range of motion of the glenohumeral joint due to various primary or secondary causes, clinically characterized as pain around the shoulder, with a reduced active and passive range of motion [12].

FS is a common cause of shoulder pain and disability. It affects approximately 2–4% of the general population [13]. Epidemiological surveys show that the prevalence increased linearly with age whilst incidence peaked at around 50 years old [14], more women than men, with an incidence of 3–5% in the general population [15], accounting for 42% in shoulder diseases, approximately 8% in orthopedic diseases [16]. Despite that many study show it is a self-limiting disease, a reversible condition, with a course of 1–3 years [17–19], this is an estimated time frame, many patients can still experience symptoms at 6 years [20]. If the shoulder’s adhesion is severe and is not treated in time, 6–17% of the patients will affect the healthy side within 5 years [21], which will seriously influence patients’ daily work and quality of life, and even lead to a loss of motor function of the shoulder. MUA (manipulation under anesthesia) is one of the most common surgical approaches for treating frozen shoulder [22]. It involves the passive tearing of the thickened inflamed capsule and contracted ligaments [23]. Originally performed under general anesthesia, now with the recent advancements in ultrasound technology are performed under brachial plexus or cervical nerve root blocks as well [24, 25]. It is the most cost-effective option [26, 27], with satisfactory short- and long-term results in ROM (range of motion), pain, and function [28–30].

Rotator cuff injury or tear is a common chronic pathology that can be initially asymptomatic and may remain unknown for a long period of time, varying from person to person. The prevalence of asymptomatic rotator cuff tears ranges from 8 to 46% [31, 32], and the prevalence of rotator tears concomitant with frozen shoulder ranges from 12.3 to 41.7% among all RC tears [33]. For frozen shoulder patient’s concomitant with RC injury intending to undergo an MUA operation, the fear of further iatrogenic rotator cuff tears exists. Although a few studies have shown that MUA is not associated with rotator cuff tears [34], and will not exacerbate the injury [35], clinically, many physicians still have concerns and exclude the patients for MUA if they have rotator cuff injury or tear on MRI [36].

Currently, there are many methods to treat FS in both conservative and surgical ways. The goal is to relieve pain and limited mobility, shorten the course of the disease, improve the patient's personal life quality, and increase social participation. Treatments for FS include conservative treatments such as NSAIDs, physical rehabilitation therapy, and intra-articular steroid injection, or invasive treatments such as manipulation under anesthesia (MUA), arthroscopic capsular release, and hydro-dilatation [36]. However, systematic reviews indicate that there is insufficient evidence and no agreement on which procedure is superior [37, 38]. What is more, when it comes to FS patients combined with rotator cuff injury, there is a shortage of high-quality comparative studies on these procedures.

The popularity of resonance examination has made the diagnosis of rotator cuff injury (or tear) more common. It is found the overall prevalence of tears in asymptomatic individuals is 34% by MRI [23] and 23% of full-thickness rotator cuff tears by ultrasound, with 51% of individuals over age 80 having tears [39]. This imagined diagnosis does not necessarily connect to the sudden onset of the stiffness and pain of the shoulders. In clinical practice, some doctors and patients become overly fixated on the results of magnetic resonance imaging (MRI), abandoning more simple and inexpensive treatment options like MUA, leading to overreliance on arthroscopic surgery for treatment [40]. However, while this approach can reduce pain to some extent [41], the surgery itself may worsen the stiffness of frozen shoulder due to surgical trauma [42, 43].

The Orthopedic Department of Shanghai Shuguang hospital has applied MUA since 2011. At present, more than 1000 cases of FS have been successfully treated by MUA, and the treatment has been carried out in Shanghai First People’s Hospital, Shanghai Baoshan District Central Hospital with same remarkable results.

During treatment, we have discovered that many patients with classic frozen shoulder symptoms also have, diagnosed by MRI scans, a rotator cuff injury or tear. Interestingly, these patients did not experience any symptoms related to their rotator cuff prior to their frozen shoulders’ sudden onsets, and after undergoing manipulation under anesthesia (MUA) treatment, their rotator cuff injury did not worsen. The current studies exploring FS combined with rotator cuff tears or injuries offered limited information on the treatment options available and on their relative effectiveness compared to each other [34, 44, 45]. A deeper comprehension of the available treatment methods, technical considerations, and risks would help clinicians create appropriate treatment plans for this situation. Due to the apparent lack of knowledge in this area, the objectives of this study are to address this gap by examining more available treatment modalities and their outcomes.

### Explanation for the choice of comparators {6b}

Intra-articular steroid injection is a common treatment of FS [37], providing short-term benefits [37, 46, 47], and also proving effective for rotator cuff tear [48]. It is an effective, common, affordable treatment with little direct transformation to the shoulder structure, that is why we choose it as our control group’s treatment.

## Objectives {7}

The objective of this trial is to observe the safety and efficacy of MUA, compared to intra-articular steroid injection, in the treatment of FS patients diagnosed with rotator cuff injury or tear by MRI. We hypothesize that the course of the disease can be shortened by MUA with quicker functional recovery and gain in the range of motion and a subsequent faster return to work compared to steroid injection treatment. Also, preoperative and sequent MRI and musculoskeletal ultrasounds will be applied to patients before and 6 months post the intervention to evaluate the condition of the patient’s rotator cuff injury or tear.

## Trial design {8}

This trial is a randomized, controlled, prospective, multicenter superiority trial with two parallel groups. Patients with FS accompanying MRI diagnoses as rotator cuff injury or tear are randomly divided into two groups: the trial group (with manipulation under brachial plexus block anesthesia as the main intervention) and the control group (with intra-articular steroid injection as the main intervention). All patients included in this study will undergo a program of shoulder exercises, normally commencing within 24 h after the corresponding intervention, with three sessions per day lasting for 2 weeks. The data will be collected before the intervention, 1 day, 2 weeks, 4 weeks, 12 weeks, and 24 weeks after the intervention. Randomization will be performed by a computer-generated random number table with a 1:1 allocation.

## Methods

### Study setting {9}

This trial is organized and implemented by the Department of Orthopedics and Traumatology of Shuguang Hospital in Shanghai, China, affiliated with Shanghai University of Traditional Chinese Medicine. The research is planned to be completed in three hospitals: (1) Shuguang Hospital Affiliated with Shanghai University of Traditional Chinese Medicine, (2) Shanghai First People's Hospital, and (3) Shanghai Baoshan District Hospital of Integrated Traditional Chinese and Western Medicine. All three hospitals are grade III, class A comprehensive hospitals in Shanghai, China. Shanghai First People’s Hospital is in the city center area, while Shanghai Baoshan District Hospital of Integrated Traditional Chinese and Western Medicine is located in rural areas, and Shuguang Hospital is located between urban and rural areas. All three hospitals in Shanghai are renowned for their departments of orthopedics and traumatology, and Shi's Orthopedics and Traumatology Department of Shuguang Hospital collaborates frequently with the other two hospitals on scientific research projects. By utilizing a practical trial design and involving a broad range of clinicians, the study’s results will be both applicable and generalizable.

### Eligibility criteria {10}

#### ***Inclusion criteria*** [49]

1. Age 40–70 years old [50]
2. Meet the diagnostic criteria for shoulder coagulation, with significantly limited shoulder range of motion (less than 100° in forward flexion, less than 10° in external rotation, and less than L5 level in internal rotation) [12] and an MRI diagnosis of rotator cuff injury (conducted by professional radiologists from the Imaging Department).
3. Voluntarily participate in this experiment and sign the informed consent

#### Exclusion criteria [49]

1. Patients who are allergic to anesthetic drugs or have skin damage, ulceration, or infection at the anesthesia puncture site, and have long-term fever symptoms
2. Patients with severe osteoporosis
3. Coexists with other serious heart, liver, kidney, and other important organic diseases and blood diseases
4. Patients with obvious upper limb, neck, and shoulder blood vessel or nerve injury or fracture
5. Pregnant or lactating women
6. Those who are currently participating in other clinical trials
7. Those who suffer from mental and psychological diseases and cannot cooperate

#### Shedding criteria

1. After inclusion, the treatment was not completed according to the planned protocol;
2. Serious adverse events occur during the treatment and, hence, cannot continue;
3. The patient’s symptoms worsened, and it was not suitable to continue participating in the trial.

#### Eligibility criteria for physicians performing the interventions:

1. The physicians must have participated in Good Clinical Practice (GCP) training.
2. They should hold a valid Chinese National Vocational Physician Certificate in Orthopedics.
3. The physicians must be licensed to practice medicine legally in China.
4. The physicians must have the qualification to conduct MUA in their respective hospitals.

### Interventions {11a}

#### Intervention description

Eligible patients will be randomized in equal proportions between the trial group (manipulation under anesthesia) and the control group (intra-articular steroid injection), undergoing a corresponding operation on day 1 and a sequent physical exercise for 14 days.

##### Trial group

The MUA will be performed by several physicians from the three hospital settings. These physicians are highly experienced and senior practitioners, with extensive backgrounds in conducting MUA for many years within their own hospitals. Additionally, prior to commencing the trial, all participating physicians underwent rigorous training with three standard operating procedure (SOP) sessions to ensure uniformity and consistency in the procedure. The MUA will be performedin the operation room to ensure safety and sterility. Before the operation, the patient needs to fast for at least 6 h. During the operation, the patient will continuously inhale oxygen at a low flow rate (2–2.5 L/min), open the intravenous infusion channel, and monitor the patient's vital signs in real-time with ECG monitoring.

A professional anesthetist of corresponding hospitals applies the brachial plexus block anesthesia in the operation room. The specific procedures are as follows: the patient lies down in a supine position, the head turns to the unaffected side, and a soft pillow is placed under the neck and shoulder to fully expose the anesthesia puncture site. After routine disinfection, the intermuscular groove formed by the sternocleidomastoid muscle and the anterior and middle scalene muscles, located under ultrasound guidance, is the entry point, where 10 ml (or 75 mg) ropivacaine + 10 ml 2% lidocaine is injected to block the brachial plexus. After 15–20 min of anesthesia, manual release begins.

##### Trail group: manipulation under anesthesia

① The patient takes a supine position. While the operator stands on the affected side of the patient, lifts the affected limb to 180° in flexion, holds the elbow of the affected side with one hand during the operation, and places the other hand on the upper end of the humerus near the humeral head at the patient’s axilla to prevent shoulder dislocation. Repeat three times when the sound of the adhesion loosening can be heard and the feeling can be felt.
② Abduct and lift the patient’s affected limb to 180°, while also being mindful of protecting the humeral head to prevent dislocation. Repeat three times.
③ Flex the affected side shoulder joint to 90° in forward flexion, bend the elbow, and internally rotate the affected side shoulder joint to 45°. Repeat three times when the sound of the adhesion loosening can be heard and the feeling can be felt.
④ Rotate the affected shoulder in the neutral position, the 45° abducted position, the 90° abducted position, the 135° abducted position, and the straight-elbow position of the glenohumeral joint. Repeat three times.
⑤ The patient lies on the healthy side, with the shoulder joint extended, internally rotated, and the elbow flexed so that the patient’s fingers can touch the healthy scapula. Repeat three times. After the operation, immobilize and protect the affected limb.
⑥ After the operation, in the ward and with the patient under continued anesthesia, release the affected limb every hour for two times to achieve complete release of the shoulder joint.

*Note:* During the operation, pay attention to enhancing communication with the patient and closely monitoring the patient’s vital signs. The technique should be gentle to avoid the possibility of complications such as fractures due to improper operation.

##### Control group: intra-articular steroid injection

The intra-articular steroid injection procedure are as follows: Thoroughly sterilize the affected shoulder, inject 2 ml of chitosan and 2.5 ml of Diprospan diluent into the posterolateral approach of the shoulder joint cavity, and inject 2.5 ml Diprospan into the supraspinatus tendon, subacromial bursa, and long head of biceps tendon, respectively.

##### Post-procedural physiotherapy (PPP)

Patients from both groups will initiate a 14-day functional exercise, starting on the first day after the intervention treatment. A uniformly and strictly trained physician guides the first functional exercise of the patients until the patients and their accompanying family members have mastered the correct exercise method. They can record relevant videos, and family members can assist and supervise patients in the initial exercise stage. Physicians follow up with patients regularly to ensure the standardization and correctness of exercise posture.

The specific methods are as follows:

① Forward elevation: The patient stands facing a wall with their feet shoulder-width apart and toes touching the wall. The fingers of the affected arm are spread out and placed on the wall. Slowly elevate the arm along the wall until the limit of pain tolerance is reached. The unaffected arm can assist in lifting the affected arm, or another person can push the scapula of the affected side from behind to help raise the arm to the maximum position.
② Forward flexion with shoulder support: The affected shoulder is flexed to 90° with the elbow flexed. The palm of the unaffected hand is gradually placed on the elbow, moving it towards the midline to bring the affected arm to rest on the opposite shoulder joint.
③ Extension with internal rotation: The patient sits or stands with the palm of the affected hand facing backward and extends the shoulder joint. Within the limit of pain tolerance, gradually rotate and adduct the affected arm while bending the elbow until the fingers touch the opposite scapula. The unaffected hand can assist the affected hand in completing the exercise.

Repeat the above movements after the patient’s pain is relieved. Perform three sets of each exercise, three times per day.

The ROM, CMS, MPQ, VAS, and PPI numbers or scores will be collected at 1 day, 2 weeks, 4 weeks, 12 weeks, and 24 weeks after the intervention. Additionally, imaging examinations will be conducted during the 24-week visit. Due to the nature of the physical examinations required, all follow-up time points must be completed by the patients at the hospital’s outpatient or inpatient departments.

### Modifications{11b}

The study allows participants to exit voluntarily, or they may be removed by study clinicians if they experience negative effects related to the study or meet the shedding criteria during the treatment. If a participant leaves due to negative effects, the study clinicians will assess if they need further treatment. Safety data will be collected for all participants who leave the study. Patients from both groups may receive additional treatment after their exit. Patients who experience severe pain and cannot tolerate MUA therapy or show no improvement within a short period of time, impacting their quality of life and sleep, should receive emergency medication such as the use of painkillers, muscle relaxants, or other medications which are deemed absolutely necessary for the participant’s well-being and then be removed from the trial.

### Adherence {11c}

Due to the nature of this study, since the main treatment is done all at one time, its adherence is guaranteed. The follow-up time points in this study are 1 day, 2 weeks, 4 weeks, 12 weeks, and 24 weeks after the patients receive the treatment. Because of the physical examination involved, all follow-up time points must be completed by the patients at the hospital's outpatient or inpatient departments. The study staff will contact participants over the phone or on WeChat to supervise their home exercise program and to make appointments before their scheduled follow-up examinations. Finally, participants will receive reimbursement for their time and transportation as 50 yuan for each visit.

### Concomitant care {11d}

The care of a patient awaiting surgery may involve administering pain relief medication to alleviate discomfort, providing guidance on how to properly take care of the affected arm (e.g., weight bearing on the upper limbs), and offering general advice on preventing additional stiffness in the limb. This will not include a specific home exercise program, as it is considered as an active intervention. After the surgery, the use of painkillers, muscle relaxants, and other medications is prohibited during the trial period. However, if there are specific circumstances such as intolerable pain, emergencies or unforeseen medical conditions, and any other situation, deemed necessary by the treating physician to ensure the patient's health, where these drugs are absolutely necessary, they must be accurately recorded on the medication registration form. Patients who had pre-existing medical conditions before enrollment will continue with their initial treatment, and any medication administered for pain relief will be noted in the medication record form. If a patient drops out of the trial or has poor compliance, the detailed reasons for this will be recorded in the case observation form, and efforts will be made to contact the patient frequently to obtain their understanding and support. Any efficacy-related measures that can be completed will be recorded, and the final treatment time will be noted. The patient’s compliance and adverse reactions will be described statistically and compared between the two groups.

### Ancillary and post-trial care {30}

Both MUA (manipulation under anesthesia), intra-articular injection, and physical exercises are routine treatments for frozen shoulder (FS) in these three hospitals. Therefore, post-trial care, which includes ongoing treatment for a frozen shoulder, is accessible to all trial participants. If a trial participant wishes to make a formal complaint, they are suggested to do this through the Medical Affairs Office in the respective hospital. If patients experience severe postoperative complications, are harmed due to research personnel’s negligence, or encounter other adverse events, they will receive appropriate medical treatment, including surgical intervention if necessary. After treatment, a satisfactory compensation plan will be discussed with the patients. If they are still not satisfied, they may have grounds for legal action against the hospital and the researchers.

### Outcomes {12}

#### Primary outcome measures

##### Comprehensive efficacy evaluation (total effective rate)

Assessment was made primarily with reference to measurements of external range of motion of the glenohumeral joint and improvement in pain.

The specific judgment is as follows:

① Cured: Shoulder pain disappears, forward flexion>150°, abduction>120°, extension>45°, and internal and external rotation>60°.
② Effective: Shoulder joint pain basically disappears, with forward flexion from 120° to 150°, extension from 30° to 45°, abduction from 90° to 120°, and external and internal rotation from 45° to 60°.
③ Improved: Shoulder pain is reduced, and the range of motion is increased compared with that before treatment, but there is still limitation of movement.
④ Ineffective: no change in indications and symptoms before and after treatment.

The total effectiveness rate = (cure + effective + improved) / number of patients × 100%

#### Constant–Murley Score (CMS)

The CMS provides an assessment of both Individual parameters and clinical symptoms, which is sufficiently sensitive to reveal even small changes in function [51]. The score consists of four domains [52]: pain (15 points), activities of daily living (20 points), movement (40points), and strength (25points). The patients self-assess the pain and the first three questions of activities of daily living, and all other items are examiner assessed.

#### Secondary outcome measures

##### Active and passive range of motion (ROM)

The forward flexion, abduction, and external rotation, in both active and passive modes, are measured by a goniometer(measuring range: 0–360°, resolution: 0.05°) . The smallest unit of measurement is 5°. Forward flexion and abduction are measured in the standing position, external rotation measured with the arm post or held at the side, and the elbow in 90° flexion. Internal rotation is estimated to which height the patient can reach on his back with the tip of the thumb, recorded in both active and passive. The vertebra level of the sacrum is recorded as 1 point. From the fifth to first lumbar vertebra, it is numbered serially 2–6 and so on.

##### Short-form McGill Pain Questionnaire

The McGill Pain Questionnaire is a multi-dimensional tool for pain assessment, for its qualitative description of pain, its publication in 1975 represented a major evolution in pain research [53]. The short-form McGill Pain Questionnaire (SF-MPQ) is a shorter version of the original MPQ and was developed later in 1987 [54], composing of three components. The first one is the pain rating index, which has 2 subscales: a sensory subscale with 11 words, and an affective subscale with 4 words from the original MPQ. These words or items are ranked on an intensity scale as 0 = none, 1 = mild, 2 = moderate, and 3 = severe. The second one is a 10-cm visual analogue scale (VAS) for average pain, and the third one is for present pain intensity [55], which are rated as 0 = no pain, 1 = mild, 2 = discomforting, 3 = distressing, 4 = horrible, and 5 = excruciating.

#### Other observation indicators

##### Patent's satisfaction with the efficacy of treatment

Including: Extremely satisfied, satisfied, average, dissatisfied, extremely dissatisfied.

##### Safety evaluation

Adverse effects, including humeral shaft fracture, glenoid fracture, shoulder dislocation, complex regional pain syndrome, neurovascular lesions, and infection, will be recorded at any time during the trial. In addition, an MRI of the glenohumeral joint and a musculoskeletal ultrasound of the shoulder will be performed 6 months after treatment to assess the safety effects of the MUA on the rotator cuff.

### Participant timeline {13}

Patients with FS will be randomly divided into the trial group and the control group. All patients included in this study will receive their disseminated treatment on Day 1 and undergo a program of shoulder exercises, for 2 weeks. The relevant data will be collected before the intervention, 1 day, 2 weeks, 4 weeks, 12 weeks, and 24 weeks after the intervention, while safety evaluations and adverse events will be recorded each visit. The specific participant’s timeline is shown in Table 1.

**Table 1:** Participant’s timeline

Timepoint (state unit)	−7~0 DAY	Treatment day	1 day	2 weeks	4 weeks	12 weeks	24 weeks
Visit number:	1	2	3	4	5	6	7
Enrollment:							
Eligibility screen	x						
Informed consent	x						
Allocation	x						
Imagining examination	x						x
Baseline questionnaire	x						
Interventions:							
Intra-articular steroid injection		x					
Manipulation under anesthesia		x					
2-week shoulder exercises			x	x			
Assessments:							
Active range of motion		x	x	x	x	x	x
Passive range of motion		x	x	x	x	x	x
CMS		x	x	x	x	x	x
MPQ		x	x	x	x	x	x
VAS		x	x	x	x	x	x
PPI		x	x	x	x	x	x
Total effectiveness rate							x
Safety evaluation		x					x
Adverse event		x					x

### Sample size {14}

The total effective rate is the primary outcome parameter. This is based on the efficacy of previous studies on MUA [56] and intra-articular steroid injection [57] in the treatment of FS. The sampling ratio for the treatment and control groups is 1:1, and the number of test subjects required for each group sets as *n*, using a two-sided test. πT and πC are the reference values for the efficacy of previous studies on MUA and steroid substance shoulder cavity injection for FS, respectively [56, 57], setting πT = 0.862 and πC = 0.428. Based on these parameters, we calculated a sample size of 160 subjects per group with a power of 80%, alpha 0.05, and a 10% drop out rate.

### Recruitment {15}

According to the sample size calculation, this trial requires 320 patients in total. This project uses online media promotion, bulletin board in the hospital, recruits from outpatient visits, and other ways to recruit subjects with stiff shoulders in the Orthopedic Department Outpatients of Shuguang Hospital, Shanghai First People's Hospital, and Shanghai Baoshan District Hospital of Integrated Traditional Chinese and Western Medicine. The participants will undergo two initial diagnostic screenings conducted by clinicians at each research center. The clinicians responsible for diagnosis and screening are unaware of the random grouping. At the scheduled clinical visit, the study clinicians will briefly explain the study to the participants. If interested, the clinicians will assess whether the participant meets the eligibility criteria. If the participant is eligible and agrees to participate, the clinicians will obtain informed consent and enroll the participant. Before signing the informed consent, participants can discuss the study details with the clinicians and ask questions. After enrollment, full-time research assistants will conduct physical and imaging examinations and assign the treatment based on randomization.

### Allocation {16a} {16b}{16c}

Eligible subjects who meet the test and research criteria will be randomly assigned into two groups, the trial group and the control group, in a 1:1 ratio. The random number table used for the randomization process is generated by statisticians at Shanghai University of T.C.M. and is not accessible to anyone else involved in the study. The randomization will be stratified by participating site and done in blocks of four. Each subject can only register and be randomized once, and their information cannot be deleted from the database. The clinicians will identify patients with frozen shoulder and confirm their eligibility using a CRF completed by a designated individual in the shoulder team. The recruiting clinician will then contact the statistical staff for randomization. The hospital staff will be informed of the allocation, but not the patient. The patient will be fully informed about the allocated therapy and given an information sheet to review before deciding to consent, which can take up to a week. If the patient does not consent, their reason for not doing so will be briefly recorded in a CRF. Those who consent will complete baseline forms and receive further treatments.

### Blinding (masking)

#### Blinding mechanism {17a}

Due to the nature of the intervention, comparing MUA and intra-articular injection treatment options, it is not possible to blind the clinicians to the allocation of treatment. However, they are strongly advised not to reveal the participant’s allocation status during follow-up assessments. An employee outside the research team will enter data into separate datasheets to ensure that the researchers who are responsible for the analyzation of the data do not have access to the information about allocation. The assessments of clinical recovery will be carried out by an experienced clinician who has undergone extensive training and is blind to treatment allocation.

#### Emergency unblinding {17b}

In this study where researchers are aware of the patients’ treatment status, emergency unblinding is typically not necessary for the researchers. However, it may be required in rare instances where the patient’s safety or well-being is at risk. The decision to unblind a patient will be made by the principal investigator in consultation with the treating physician and an independent data and safety monitoring board. An independent, unblinded investigator who is not involved in the patient’s care or data analysis will carry out the unblinding process, confirming the patient’s identity and the reason for unblinding on the case report form. The study team will review the unblinding and make any necessary adjustments to the patient’s treatment plan or report adverse events to regulatory authorities. It is important to limit the use of emergency unblinding and minimize its impact on the study results.

### Data collection {18a}

#### Trial procedures and evaluations

**Fig. 1:**
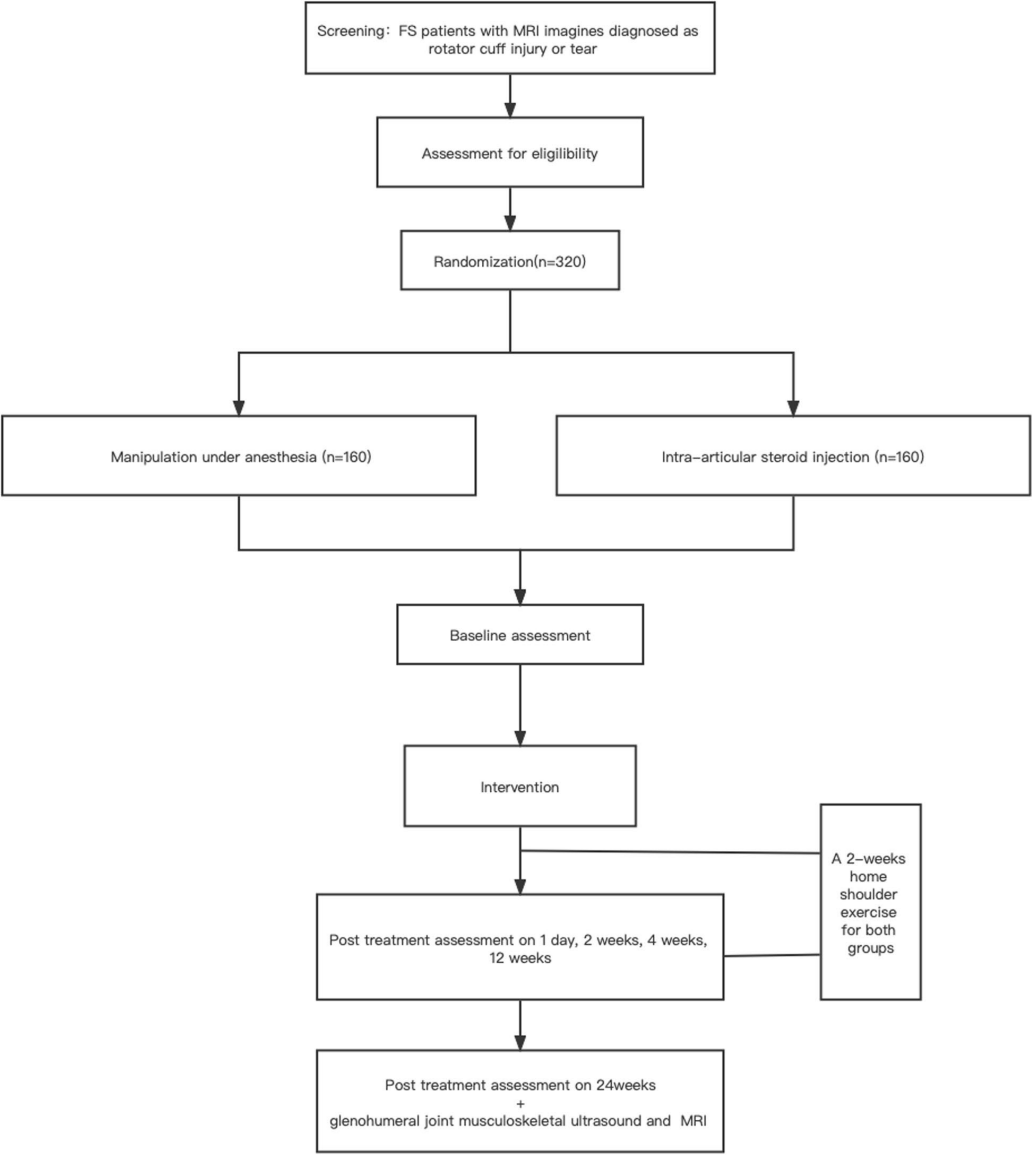
Trial flowchart

Patients with FS accompanying MRI diagnoses as rotator cuff injury are randomly divided into two groups: the trial group (with manipulation under brachial plexus block anesthesia as the main intervention) and the control group (with intra-articular steroid injection as the main intervention). All patients included in this study will undergo a program of shoulder exercises, normally commencing within 24 h after the corresponding intervention, with three sessions per day for 2 weeks. The data will be collected before the intervention, 1 day, 2 weeks, 4 weeks, 12 weeks, and 24 weeks after the intervention. Because of the physical examination involved, all follow-ups will be done in the outpatient or inpatient department of the hospital. The investigators used a Case Report Form (CRF) to collect real-time clinical data from the subjects, and any modifications to a CRF will be signed and dated. The collection of outcome data is accomplished through clinical evaluations that are conducted by either a trained practitioner, an orthopedic physician, or a resident in the orthopedic inpatient or outpatient setting. All personnel will undergo training to ensure data quality. The subjects’ data will be securely stored at a designated location. The information collected during the research will be treated as confidential and accessible only to the researchers. The schedule of enrolment, interventions, and assessments is depicted in Fig. 1.

### Retention {18b}

Due to the nature of this study, since the main treatment is done all at one time, its adherence is guaranteed. The follow-up time points in this study are 1 day, 2 weeks, 4 weeks, 12 weeks, and 24 weeks after the patients received the treatment. Because of the physical examination involved, all follow-up time points must be completed by the patients at the hospital’s outpatient or inpatient departments. The study staff will contact participants over the phone or on WeChat to supervise their home exercise program and to make appointments before their scheduled follow-up examinations. Finally, participants will receive reimbursement for their time and transportation as 50 yuan for each visit.

### Data management {19}

The investigators used a Case Report Form (CRF) to collect real-time clinical data from the subjects. To ensure the objectivity of the clinical data, all subjects in this study are followed up and observed by the same investigator. The CRF form needs to be filled and reviewed at the same time when examining the patients. When finally entering the data, double entry and cross-checking will be used, and data will be periodically and randomly checked by data management staff to ensure the accuracy. A special statistical staff at Shanghai University of T.C.M. will analyze the data unaware of the allocation.

## Statistical methods

### Outcomes {20a}

The data will undergo encoded analysis and various patient details, such as their baseline information, range of motion, CMS, VAS, MPQ, PPI, duration of symptoms before treatment, and past histories, will be compared and summarized between the groups. The outcome variables of all patients will also be summarized by treatment group and time, following descriptive statistics such as the number of patients, mean and standard deviation (or median and interquartile range) for continues variables, and relative frequencies for categorical variables. The number of participants included in each analysis will be reported, and we will adhere to the intention to treat principle regarding the group assignment.

Differences between the various treatments will be calculated using one-way ANOVA statistics; the follow-up data of each group will be statistically analyzed using ANOVA of repeated measures if they conformed to normal distribution and chi-square, and if they do not, the rank sum test will be used; logistic regression will be used to analyze the correlation between each treatment method and the efficacy. The study will categorize complications by types, such as infection, post-operative pain, nerve, blood vessel, tendon, or bone injury, and count them overall. Poisson regression analysis will be used with corrections for overdispersion when appropriate. The impact of treatment on complication rates will be expressed as MUA to intra-articular injection rate ratios. The safety of the treatment will be evaluated by identifying and summarizing any adverse events that occur during the study. All treatment effects will be reported with their 95% confidence intervals and *p*-values. The statistical analysis will be conducted using the IBM SPSS statistical package (version 25.0).

### Additional analyses {20b}

Based on the clinical observation indexes and shoulder joint MRI and musculoskeletal ultrasound imaging data before and after treatment of a total of 320 patients collected in the study, a multi-factor correlation analysis will be performed using subgroup analysis, sensitivity analysis, and logistic regression so that the following can be determined.

1.1 Whether there was a positive correlation between the shoulder joint active and passive mobility, pain and the data of the staging, and degree of rotator cuff injury characterized by shoulder MRI and musculoskeletal ultrasound imagining. During the MRI analysis, the primary analysis indicator used to assess the rotator cuff injury was the Ellman classification system [58], which takes into account both the tear's location and depth. There are two types of tears: partial-thickness tear (P) and full-thickness tear (F). For partial-thickness tears, they are categorized as follows: A: articular, B: bursal, C: intratendinous, and further graded as Grade 1 (<3 mm), Grade 2 (3−6 mm), and Grade 3 (>6 mm). For full-thickness tears, they are categorized based on the affected muscles: (A) supraspinatus, (B) infraspinatus, (C) teres minor, and (D) subscapularis and graded as Grade 1 (small, <2 cm), Grade 2 (large, 2–4 cm), Grade 3 (massive, >5 cm), and Grade 4 (cuff arthropathy). Since one of the most common complications of a rotator cuff tear is supraspinatus muscle atrophy [59], and fat infiltration is correlated with the severity of the rotator cuff tear [60], the secondary indicators will be Goutallier’s classification [61, 62] on fatty degeneration of cuff muscles and the Thomazeau classification of supraspinatus muscle atrophy [63]. Our designated medical professional will be responsible for interpreting all the MRI findings and comparing MRI images in later visits. This ensures integrity and accuracy in data analysis, minimizing the potential errors in data interpretation.
1.2 The type and the degree of rotator cuff injury for which the MUA is suitable, and the best indication for the maneuver.
1.3 Whether there is an increase in the degree of rotator cuff injury after MUA and to verify the safety of MUA on patients with rotator cuff injury.

### Analysis population and missing data {20c}

If a case falls off or the compliance of a subject is poor, the reasons for this occurrence must be documented in detail on the case observation form. The patient will be contacted in time to obtain his/her understanding and support. Any evaluation items that can be completed should still be completed, and the date of the last treatment must be recorded. The cases where the patient's treatment was discontinued, poor compliance, and adverse reactions will be described statistically and compared and evaluated between the groups.

#### Plans to give access to the full protocol, participant level-data and statistical code {31c}

The study protocol is publicly available on the website Chictr.org.cn with registration number: ChiCTR2200067122. The trial datasets generated or analyzed during the current study during the study are not publicly available. Patient data that can identify individuals will only be shared within the clinical team on a need-to-know basis to ensure proper clinical care and appropriate follow-up.

## Data monitoring

### Formal committee {21a}

The study will be supervised by the Data and Safety Monitoring Board (DSMB), which consists of physicians, ethicists, medical statisticians, radiologists, sonographers, and a clinical manager. Their responsibility is to oversee the study at regular intervals, every 3 months. The DSMB will examine safety data and clinical effectiveness reports and determine if the clinical trial should continue. The primary investigator at Shanghai University of TCM will receive their feedback.

### Interim analysis {21b}

During the project’s midway point, it is necessary to review and amend the project research standard operating procedure (SOP), investigator manual, and case report form. Detailed training and explanation of the test scheme, research operation SOP, and investigator manual contents should be provided during work training and summary meetings. A report on the enrolment progress, treatment success rates, adverse events, and protocol deviations should be given to the Data Safety Monitoring Board members who are not involved in experimental research and treatment. The Data Safety Monitoring Board (DSMB) members will have access to the interim analysis results. These results will be presented to the DSMB in a secure and confidential manner to maintain data integrity and participant confidentiality. The DSMB will thoroughly assess the interim analysis findings, including safety data, treatment efficacy, and any emerging trends, to evaluate the trial's progress. The Data Safety Monitoring Board (DSMB) has the rights to make the final decision on whether to continue or terminate the trial. The DSMB will consider participant safety, ethical considerations, and the overall scientific validity of the trial's outcomes in making this critical determination.

### Safety/harms {22}

Throughout the trial, it is necessary to monitor and record any adverse events and reactions. If adverse events occur, such as negative impacts on patients' consciousness, emotions, physical activity, sleep, blood pressure, heart rate, respiration, upper limb fractures, skin damage, nerve injury, bleeding, and black stools, they will be documented thoroughly on the case report form. This documentation should include the time, severity, duration, and treatment measures, as well as an analysis of the correlation between the adverse reaction and the experimental treatment method, considering any complications and concurrent treatments. The clinical observe physician can decide to halt the trial if the patient's condition warrants it. Any patients who discontinue treatment due to adverse reactions should be closely monitored and investigated, with detailed records of the results.

### Auditing {23}

The Shanghai University of TCM research team is directly overseeing and managing this project. They have created an online WeChat group for research supervision, along with a special case inspector from Shuguang Hospital, which is responsible for the project. Additionally, the supervisor of Shuguang Hospital and the leaders of each center have established a WeChat working group. They have the ability to conduct spot checks and audits on the completeness and accuracy of the case report forms filled out at each center, at any time. The frequency for auditing is once every 3 months. During the auditing process, procedures are followed to review and assess the relevant activities, records, and systems to ensure compliance, accuracy, and adherence to established guidelines and standards. The auditing team will thoroughly examine the data, processes, and documentation to identify any potential issues, discrepancies, or areas for improvement. The findings from the audit will be documented and appropriate actions will be taken to address any identified concerns and ensure ongoing compliance and quality.

### Protocol amendments {25}

If any changes are made to the study protocol that may impact the study's conduct, benefit to the patient, or patient safety, including modifications to study objectives, design, patient population, sample size, study procedures, or significant administrative aspects, a formal amendment to the protocol will be required. The Shanghai Science and Technology Commission must agree on this amendment, which will then require approval from the Shuguang Hospital Ethics Committee before implementation, and the health authorities will be notified in accordance with local regulations.

### Informed consent process {26a}

All participants meet the inclusion criteria and agrees to participate will receive an information sheet and consent form. Before signing the informed consent, participants can discuss the study details with the clinicians and ask questions and take as much time as they need to consider their decision. The researchers conducting the study will obtain informed consent from the participants. All enrollment in the study will be subject to obtaining a signed consent form beforehand. After enrollment, full-time research assistants will conduct physical and imaging examinations and assign the treatment based on randomization. The results files of the participants will be coded, and personal data will not be disclosed.

### Confidentiality {27}

The research center for the project will keep the medical records of the participants, such as the CRF and MRI results, fully secured and stored. Confidentiality of the personal data gathered during the experiment will be maintained, and all data will be saved on an encrypted computer located in the research center. Access to this computer will only be granted to authorized researchers working on the project, and the data will only be used for the purposes of the project and not for any other reasons.

## Discussion

In this study, we propose to compare the efficacy of manipulation under anesthesia versus intra-articular steroid injection in the treatment of patients diagnosed with rotator cuff injury with frozen shoulder. We expect that the MUA group will have a superior outcome compared to the intra-articular steroid injection group in terms of the primary outcome measure. If our hypothesis is confirmed, this study could lead to a change in the standard of care for patients with rotator cuff injury and frozen shoulder and provide a new treatment option with fewer side effects and better outcomes.

It is important to note that this study has several limitations. First, the study is single-blinded due to the nature of the interventions, meaning that the physicians will not be blinded to the treatment group, which could introduce bias. Second, the study is limited to patients diagnosed with rotator cuff injury with frozen shoulder based on MRI findings and may not be generalizable to patients with other types of shoulder injuries. Finally, the study is limited to 24 weeks of follow-up, and longer-term outcomes are not evaluated.

In conclusion, this study protocol outlines a randomized controlled trial that will compare the efficacy of manipulation under anesthesia versus intra-articular steroid injection in the treatment of patients diagnosed with rotator cuff injury with frozen shoulder. The study has the potential to provide important insights into the treatment of this common condition and could lead to improvements in patient care.

## Dissemination policy

### Trial results {31a}

The collected results of the research will be presented in scientific conferences at a national or international level, and also submitted for publication in academic journals that are reviewed by experts in the field.

#### Authorship

The authors of present study protocol are as below:

Wuwei Song, Xiaoyu Guo, Xiang Wang, Jiacheng Yu, Wenyu Jiang, Chen Wei, and Yuhao Zhao.

## Study administration

### Sponsor and funder

The funding body plays no role in the design of the study and collection, analysis, and interpretation of data and in writing the manuscript.

### Trial committees {5d}

We have developed the following committees for the trial. Study principal investigator: Xiang Wang. Steering committee: Xiang Wang (chair), Hongsheng Zhan, and Yuelong Cao. The Steering Committee will provide strategic oversight and guidance for the trial. Chaired by Prof. Xiang Wang, including key investigators, Hongsheng Zhan and Yuelong Cao, who will contribute their expertise in orthopedics and trial procedure. Methods Center staff: Xiang Wang, Xiaoyu Guo, Wuwei Song, Wenyu Jiang, and Jiacheng Yu. This team will be responsible for data collection, storage, and quality control. They will work closely with the Coordinating Center to ensure that data are accurately captured and securely maintained throughout the trial. Data monitoring committee: Yongfang Zhao (chair), Zhengyan Li, Ting Liu, and Guoqing Du. This committee will ensure that data are captured accurately and in a timely manner. Site audit committee: Hongsheng Zhan, Zheng Deng, and Kaiyong Zhang. The Site Audit Committee will oversee the conduct of the trial at individual investigational sites to ensure compliance with the study protocol and regulatory requirements.

### Patient and public involvement

No patient involved.

## Trial status

Protocol version 1.0, which was revised on 11 November 2022. Recruitment began on 1 Jan 2023, and the approximate date for the completion of recruitment will be 31 December 2024.

## Data Availability

The data generated during the trials and CRFs are only accessible by the research team. They are not accessible to the public owing to privacy protection laws in China. However, interested parties may obtain the datasets by making a reasonable request to the corresponding author.

## Abbreviations

CMS: Constant–Murley Score
CRF: Case Repot Form
FS: Frozen shoulder
MPQ: McGill Pain Questionnaire
MUA: Manipulation under anesthesia
PPI: Present pain intensity
PPP: Post-procedural physiotherapy
ROM: Range of motion
RC: Rotator cuff
SOP: Standard operating procedure
VAS: Visual analog scale
